# Acupuncture for hearing loss after traumatic brain injury: A protocol for systematic review of randomized controlled trial

**DOI:** 10.1097/MD.0000000000016553

**Published:** 2019-07-26

**Authors:** Wei-feng Wang, Lin-hong Yang, Hai-jiang Yu, Shu-hong Zhang, Jian-qi Xiao

**Affiliations:** a Department of Neurosurgery; b Department of Otorhinolaryngology, First Affiliated Hospital of Jiamusi University; c Department of Biology, Jiamusi University School of Basic Medicine, Jiamusi; d Department of Neurosurgery, The First Hospital of Qiqihar City; e Affiliated Qiqihar Hospital, Southern Medical University, Qiqihar, China.

**Keywords:** acupuncture, effectiveness, hearing loss, randomized controlled trial, safety, traumatic brain injury

## Abstract

**Background:**

This study aims to systematically assess the effectiveness and safety of acupuncture on hearing loss (HL) after traumatic brain injury (TBI).

**Methods:**

In this study, the following databases will be retrieved from inception up to the May 1, 2019: PUBMED, EMBASE, Cochrane Library, Cumulative Index to Nursing and Allied Health Literature, Allied and Complementary Medicine Database, and Chinese Biomedical Literature Database. All databases will be retrieved without any language restrictions. Two reviewers will independently carry out article selection, data collection, and risk of bias evaluation. Any disagreements will be solved by a third reviewer through discussion.

**Results:**

This study will systematically investigate the effectiveness and safety of acupuncture for treating HL after TBI through evaluating HL assessment, hearing threshold, quality of life, and adverse events.

**Conclusion:**

The expected findings of this study will provide latest evidence for assessing the effectiveness and safety of acupuncture for HL after TBI.

**Ethics and dissemination:**

This study is supposed to be published in a peer-reviewed journal. No ethical approval is needed because this study will based on the literature analysis, but not the individual patient.

**PROSPERO registration number:**

PROSPERO CRD42019133417.

## 1 Introduction

Traumatic brain injury (TBI) is a very important factor which can cause death or disability worldwide.^[1–3]^ It has been estimated that >40% of patients were hospitalized as a result of TBI.^[4,5]^ Its prevalence rates vary from 3.2 to 5.3 million in the United States.^[4,5]^ Many studies have reported that many factors can cause TBI, including motor vehicle accidents, fall from heights, and blunt trauma.^[2,6–10]^ Such disorder can result in long-term disability of cranial nerve deficit, headache, behavioral changes, hemiparesis, and hearing loss (HL).^[11–22]^

Previous studies have reported that acupuncture can treat HL effectively, including HL caused by TBI.^[23–27]^ However, presently, no study has systematically assessed its effectiveness and safety for the treatment of TBI patients with HL. Thus, this study aims to explore the effectiveness and safety of acupuncture for the treatment of HL caused by TBI.

## 2 Methods

### 2.1 Inclusion criteria for study selection

#### 2.1.1 Type of studies

This study will consider all associated randomized controlled trials (RCTs) of acupuncture for HL after TBI. However, all other studies, except RCTs, will be excluded.

#### 2.1.2 Type of participants

This study will consider all patients who are clinically diagnosed with HL after TBI without any restrictions of age, sex, and race.

#### 2.1.3 Type of interventions

The patients in the experimental group can utilize acupuncture alone. Any combinations of acupuncture with other treatments will not be considered.

The patients in the control group can use any treatments, except any forms of acupuncture.

#### 2.1.4 Type of outcomes

The primary outcome includes HL. It can be measured by any scales or tools, such as Brock grade, acceptable noise level test. The secondary outcomes consist of hearing threshold, as assessed by pure-tone audiometry, speech audiometry, or any other relevant tools; and quality of life, as measured by any associated scales, such as 36-Item Short Form Health Survey or others. In addition, we will also assess recorded adverse events.

### 2.2 Search strategy

The following electronic databases will be retrieved from inception up to May 1, 2019: PUBMED, EMBASE, Cochrane Library, Cumulative Index to Nursing and Allied Health Literature, Allied and Complementary Medicine Database, and Chinese Biomedical Literature Database without any language restrictions. The detailed strategy of PUBMED is shown in Table 1. Similar strategies will be used to any other electronic databases.

**Table 1:**
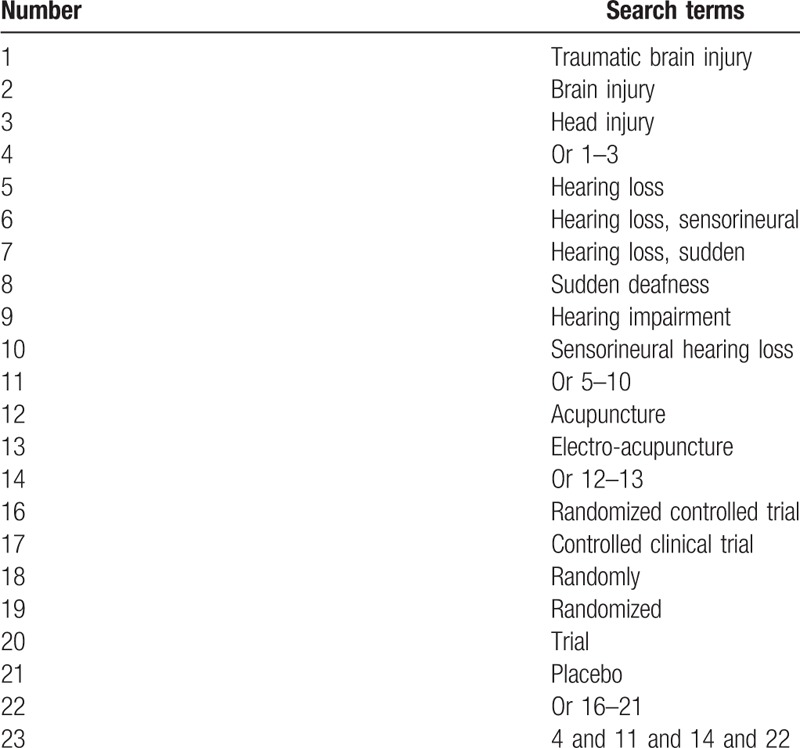
Search strategy applied in PUBMED database.

In addition, this study will also retrieve clinical trial registry, dissertations, and reference lists of all included studies.

### 2.3 Data collection and management

#### 2.3.1 Study selection

Two reviewers will independently scan the titles and abstracts for all identified literatures.

All irrelevant studies will be excluded at the first stage. After that, all remaining studies will be read by full texts at second stage. The whole process of study selection will abide to the predefined study eligibility criteria. Its results will be presented in the flowchart of Preferred Reporting Items for Systematic Review and Meta-analysis with detailed reason for each excluded study at each stage.

#### 2.3.2 Data collection

After selection, data will be collected from each eligible study based on the previous designed data collection sheet. Two reviewers will independently collect data. Any disagreements will be resolved by a third reviewer through discussion. The collected information comprise of general information, such as title, first author, location, among others; patient characteristics, such as age, sex, and so on; study methods, such as randomization, blinding, and so on; treatment details, such as dosage, frequency, and so on; outcomes, such as primary, secondary outcomes, and so on.

If some essential information is missing or insufficient, we will contact primary authors by email and request those data. If we cannot achieve those data, we will only analyze available data.

#### 2.3.3 Risk of bias assessment

In this study, we will use Cochrane risk of bias tool for the risk of bias assessment. It consists of 7 aspects, and each aspect is divided as high risk of bias, unclear risk of bias, and low risk of bias. Two reviewers independently assess all risk of bias for each eligible study. A third reviewer will judge any divergences regarding the risk of bias assessment between two reviewers.

### 2.4 Data synthesis and analysis

#### 2.4.1 Measurement of treatment effect

For continuous outcomes, the collection data will be expressed as mean difference or standardized mean difference with 95% confidence intervals. As for dichotomous outcomes, the collection data will be expressed as risk ratio with 95% confidence intervals.

#### 2.4.2 Assessment of heterogeneity

Heterogeneity will be checked by using *I*^2^ index. The value of *I*^2^ <50% (*I*^2^ ≤50%) means satisfied heterogeneity, and a fixed-effect model will be used. Otherwise, the value of *I*^2^ >50% means significant heterogeneity, and a random-effect model will be utilized.

#### 2.4.3 Data synthesis

RevMan 5.3 Software will be used for statistical analysis in this study. If *I*^2^ is <50%, data among eligible studies will be pooled and meta-analysis will be carried out. Otherwise, data pooling and meta-analysis will be carried according to the results of subgroup analysis. If significant heterogeneity still exists after subgroup analysis, data will not be pooled, and only narrative summary will be reported.

#### 2.4.4 Subgroup analysis

If the value of *I*^2^ is >50%, subgroup analysis should be performed to detect any possible reasons for the significant heterogeneity according to the different characteristics, treatment schedules, and outcome measurements.

#### 2.4.5 Sensitivity analysis

Sensitivity analysis will also be carried out to check the robustness of pooled results data, missing data, and methodological quality.

#### 2.4.6 Reporting bias

If sufficient studies are included, we will also carry out funnel plot and Egger regression test to identify any possible reporting bias.

## 3 Discussion

Numerous clinical studies have reported that acupuncture can be utilized for the treatment of patients with HL caused by TBI.^[23–27]^ This study will comprehensively search as more as possible electronic databases to avoid missing more potential studies. Two reviewers will independently carry out all literature identification, data extraction, and methodological quality assessment. Any divergences will be solved by a third reviewer via discussion. The results of this study will summarize the effectiveness and safety of acupuncture for the treatment of HL caused by TBI. It will provide very helpful evidence for clinical practice and patients.

## Author contributions

**Conceptualization:** Wei-feng Wang, Hai-jiang Yu, Shu-hong Zhang, Jian-qi Xiao.

**Data curation:** Wei-feng Wang, Lin-hong Yang, Hai-jiang Yu, Jian-qi Xiao.

**Formal analysis:** Wei-feng Wang, Lin-hong Yang, Hai-jiang Yu, Shu-hong Zhang, Jian-qi Xiao.

**Investigation:** Lin-hong Yang, Jian-qi Xiao.

**Methodology:** Wei-feng Wang.

**Project administration:** Hai-jiang Yu.

**Resources:** Wei-feng Wang, Lin-hong Yang, Hai-jiang Yu.

**Software:** Wei-feng Wang, Hai-jiang Yu, Shu-hong Zhang.

**Supervision:** Shu-hong Zhang, Jian-qi Xiao.

**Validation:** Wei-feng Wang, Lin-hong Yang, Hai-jiang Yu, Shu-hong Zhang, Jian-qi Xiao.

**Visualization:** Wei-feng Wang, Lin-hong Yang.

**Writing – original draft:** Wei-feng Wang, Lin-hong Yang, Hai-jiang Yu, Shu-hong Zhang, Jian-qi Xiao.

**Writing – review & editing:** Wei-feng Wang, Lin-hong Yang, Hai-jiang Yu, Shu-hong Zhang, Jian-qi Xiao.
